# Influence of ambulatory blood pressure-related indicators within 24 h on in-hospital death in sepsis patients

**DOI:** 10.7150/ijms.67967

**Published:** 2022-02-07

**Authors:** Fengshuo Xu, Luming Zhang, Tao Huang, Rui Yang, Didi Han, Shuai Zheng, Aozi Feng, Liying Huang, Haiyan Yin, Jun Lyu

**Affiliations:** 1 Intensive Care Unit, The First Affiliated Hospital of Jinan University, Guangzhou, Guangdong Province, China.; 2 School of Public Health, Xi'an Jiaotong University Health Science Center, Xi'an, Shaanxi Province, China.; 3 Department of Clinical Research, The First Affiliated Hospital of Jinan University, Guangzhou, Guangdong Province, China.; 4 School of Public Health, Shaanxi University of Chinese Medicine, Xianyang, Shaanxi Province, China.

**Keywords:** sepsis, ambulatory blood pressure, in-hospital death, MIMIC-IV

## Abstract

**Background:** Sepsis is a serious public health problem worldwide. Blood pressure is one of the indicators that is closely monitored in intensive-care units, and it reflects complex interactions between the internal cardiovascular control mechanism and the external environment. We aimed to determine the impact of indicators related to the ambulatory blood pressure on the prognosis of sepsis patients.

**Methods:** This retrospective study was based on the Medical Information Mart for Intensive Care IV database. Relevant information about sepsis patients was extracted according to specific inclusion and exclusion criteria. Examined parameters included the average blood pressure, blood pressure variability (BPV), and circadian rhythm, and the study outcome was in-hospital death. We investigated the effects of these indicators on the risk of in-hospital death among sepsis patients using Cox proportional-hazards models, restricted cubic splines analysis, and subgroup analysis.

**Results:** This study enrolled 10,316 sepsis patients, among whom 2,117 died during hospitalization. All parameters except the nighttime variation coefficient of the diastolic blood pressure (DBP) were associated with in-hospital death of sepsis patients. All parameters except for fluctuations in DBP exhibited nonlinear correlations with the outcome. The subgroup analysis revealed that some of the examined parameters were associated with in-hospital death only in certain subgroups.

**Conclusion:** Indicators related to the ambulatory blood pressure within 24 h are related to the prognosis of sepsis patients. When treating sepsis, in addition to blood pressure, attention should also be paid to BPV and the circadian rhythm in order to improve the prognosis and the survival rate.

## Introduction

Sepsis is a serious public health problem worldwide. In 2016, sepsis was redefined as a life-threatening infection combined with an acute increase in Sequential Organ Failure Assessment (SOFA) score of ≥2 1. In the United States there were 751,000 sepsis patients in 1995 (3.0 per 1,000 population, 2.26 per 100 discharged patients) 2, of which 383,000 (51.1%) patients received intensive care and 130,000 (17.3%) patients were monitored and nursed in intermediate-care units.

Blood pressure is one of the indicators that is closely monitored in intensive-care units for both short-term (minutes to hours) and long-term (days to months) fluctuations. It reflects complex interactions between the internal cardiovascular control mechanism and the external environment. Blood pressure variability (BPV) reflects the degree of fluctuation of the blood pressure over a certain period of time 3. It can predict the development, progression, and severity of cardiac, vascular, and renal organ damage in patients with hypertension, as well as cardiovascular events and mortality 4-6. BPV also plays an important role in other diseases. Daily variability in the systolic blood pressure (SBP) has been shown to affect the functional prognosis of patients with acute ischemic stroke 7. Moreover, elevated BPV is associated with a poor long-term prognosis of patients with acute stroke 8. A prospective study involving 40 patients with sepsis found a positive correlation between BPV and disease severity markers (lactate and SOFA scores), indicating that BPV monitoring is of great significance for the prognosis of patients with sepsis 9. Early blood pressure changes in patients with sepsis are induced by changes in various systems, including the nervous and cardiovascular systems. Monitoring the blood pressure of patients is therefore very important when considering treatment, and the treatment plan should be continuously adjusted according to detected changes in the blood pressure of individual patients. However, few studies have investigated the relationships of sepsis with blood pressure and its variability.

The Medical Information Mart for Intensive Care IV (MIMIC-IV) is a large public database that records comprehensive clinical information about patients. We used this database to explore the impact of indicators related to the ambulatory blood pressure on the prognosis of sepsis patients in detail.

## Methods

### Data source

The data used in this study were obtained from version 0.4 of the MIMIC-IV database. This is a large, single-center, and open-access database that includes data on 257,366 admissions from 196,527 patients admitted to Beth Israel Deaconess Medical Center from 2008 to 2019 10-13. MIMIC-IV was approved by the institutional review boards of Beth Israel Deaconess Medical Center (Boston, Massachusetts, USA) and Massachusetts Institute of Technology (Cambridge, Massachusetts, USA). All information related to the identity of each patient was recoded, and all identifiable information was hidden, which removed the need to obtain informed consents.

We qualified to use the MIMIC-IV database after completing an online course from the National Institutes of Health and passing the examination on Protecting Human Study Participants (Record ID: 38455175).

### Case selection

According to Sepsis 3.0 criteria, sepsis is defined as a suspected or confirmed infection plus an acute increase of >2 points in the SOFA score 14,15. We used this definition to identify patients with sepsis in the MIMIC-IV database. If a patient had multiple occurrences of sepsis during the same hospitalization period, the analysis was performed on the first occurrence. After identifying the study population, we used Structured Query Language programming in Navicat Premium software (version 11.2.7.0) to extract information on the basis of their hadm_id and stay_id parameters. Patients who were younger than 18 years, had died within 24 h after the sepsis diagnosis, or had missing information on covariates that needed to be adjusted were excluded from this study.

### Outcome

The outcome of this study was in-hospital death, and cases in which no death was observed during hospitalization were considered censored. Follow-up began at the time of a sepsis diagnosis and ended at the time of in-hospital death or discharge. The survival time was calculated in days.

### Examined parameters

We noninvasively recorded SBP, diastolic blood pressure (DBP), and mean blood pressure (MBP) for the first 24 h after the diagnosis of sepsis. Considering that a patient may have their blood pressure measured more than once during the same hour, we used hourly averages. Daytime blood pressure was measured from 8:00 to 22:00, and nighttime blood pressure was measured from 22:00 to 8:00 the next day. The examined parameters included the following: (1) average blood pressure indicators: mean values of SBP, DBP and MBP over 24 h (SBPmean, DBPmean, MBPmean) and their corresponding daytime values (SBPmeanD, DBPmeanD, MBPmeanD) and nighttime values (SBPmeanN, DBPmeanN, MBPmeanN); (2) BPV indicators: standard deviations of SBP, DBP, and MBP over 24 h (SBPsd, DBPsd, MBPsd) and their corresponding daytime values (SBPsdD, DBPsdD, MBPsdD) and nighttime values (SBPsdN, DBPsdN, MBPsdN); coefficients of variation of SBP, DBP, and MBP over 24 h (SBPcv, DBPcv, MBPcv) and their corresponding daytime values (SBPcvD, DBPcvD, MBPcvD) and nighttime values (SBPcvN, DBPcvN, MBPcvN); and (3) circadian rhythm indicators: fluctuation in SBP [SBPF; = (SBPmeanD - SBPmeanN)/SBPmeanD × 100%] and fluctuation in DBP [DBPF; = (DBPmeanD - DBPmeanN)/DBPmeanD × 100%].

### Covariates

Any indicators that might confuse the relationship between examined parameters and outcome were adjusted as covariates in the subsequent analyses. These covariates included the following: (1) demographic indicators: age, gender, ethnicity, and body weight; (2) comorbidity indicators: Charlson Comorbidity Index, myocardial infarct, congestive heart failure, cerebrovascular disease, renal disease, mild liver disease, severe liver disease, uncomplicated diabetes, complicated diabetes, and malignant cancer; (3) laboratory test indicators: lactate, creatinine, and white blood cells; (4) disease severity scores: SOFA score and Acute Physiological Score III (APSIII); and (5) treatment-related indicators: use of mechanical ventilation, vasopressor, and continuous renal replacement therapy (CRRT). For indicators measured multiple times during hospitalization, we analyzed the first measurements made after the diagnosis of sepsis.

### Statistical analysis

SBP, DBP, and MBP are dynamic parameters, and some measured values will inevitably be missed at certain time points. In order to reduce the selection bias caused by the exclusion of too many cases with missing information, we used the Amelia package of R software to impute the missing values by the incorporation of polynomials of time to fit a model to predict missing values. The approach used meant that values observed close to the time point of a missing value greatly influenced the calculation of the missing value, while those that were not close had lower weights in the calculation model 16.

The average blood pressure indicators were divided into three categories according to the diagnostic criteria for hypertension: hypotensive group (SBP <90 mmHg, DBP <60 mmHg, MBP <70 mmHg), normal blood pressure group (90 mmHg ≤ SBP < 140 mmHg, 60 mmHg ≤ DBP < 90 mmHg, 70 mmHg ≤ MBP < 105 mmHg), and hypertensive group (SBP ≥140 mmHg, DBP ≥90 mmHg, MBP ≥105 mmHg). BPV and circadian rhythm indicators were divided into four categories according to their respective quartiles.

The Kolmogorov-Smirnov test was used to determine whether the continuous variables were normally distributed. If they were, mean and standard-deviation values were used to describe the distribution, and Student's t test was used to analyze differences between groups. If continuous variables were not normally distributed, median and interquartile-range values were used, and differences between groups were analyzed using the Mann-Whitney U test. Categorical variables were expressed as frequency and percentage values, and intergroup differences were analyzed using the chi-square test or Fisher's exact test, as appropriate.

The examined parameters were first analyzed as continuous variables, and Cox proportional-hazards models with unadjusted and adjusted covariates were constructed to analyze the relationships between the examined parameters and in-hospital death. The examined parameters were then analyzed as categorical variables, and Kaplan-Meier curves were drawn to show the survival of patients in different groups, while the log-rank test was used to analyze survival differences between groups. Similarly, Cox proportional-hazards models with unadjusted and adjusted covariates were also constructed. In addition, we added cross-product terms of examined parameters and stratification variables (age [<65, ≥65 years], gender [male, female], and usage of vasopressors [to distinguish sepsis and septic shock according to their use or nonuse) 1) to models to explore possible interaction effects. For those exhibiting significant interactions, we performed a further stratified analysis. Finally, we also used restricted cubic splines (RCS) to explore the dose-response relationships between examined parameters and in-hospital death. Models with three to five nodes were fitted, all covariates were adjusted, and the model with the minimum Akaike information criterion was selected. The likelihood-ratio test was used to examine the overall statistical associations and potential nonlinear relationships. Similarly, interaction tests and stratification analyses were performed 17. All statistical analyses were performed using R software (version 4.0.3), and a two-sided P value of >0.05 was considered statistically significant.

## Results

### Baseline characteristics

This study enrolled 10,316 patients with sepsis, among whom 2,117 died during hospitalization. The median [interquartile range] follow-up time was 10 6, 20 days. The characteristics of patients in the survival and death groups are listed in Table 1. Compared with the survival group, the death group was older (70 [59, 80] years old vs. 66 [55, 78] years old), and had higher SOFA scores (3 3, 5 vs. 3 2, 4), higher APSIII (78 [61, 98] vs. 51 [39, 66]), and more comorbidities (4 2, 6 vs. 3 1, 5). The death group also had higher rates than the survival group of using ventilation (84.2% vs. 59.4%), vasopressors (67.5% vs. 36.6%), and CRRT (16.3% vs. 3.8%).

### Cox proportional-hazards models

The results of the constructed Cox models are presented in Table 2. When each examined parameter was used as a continuous variable, SBPsdN, DBPF, and all average blood pressure indicators except DBPmeanN were correlated with in-hospital death after adjusting for covariates.

**Figure 1:**
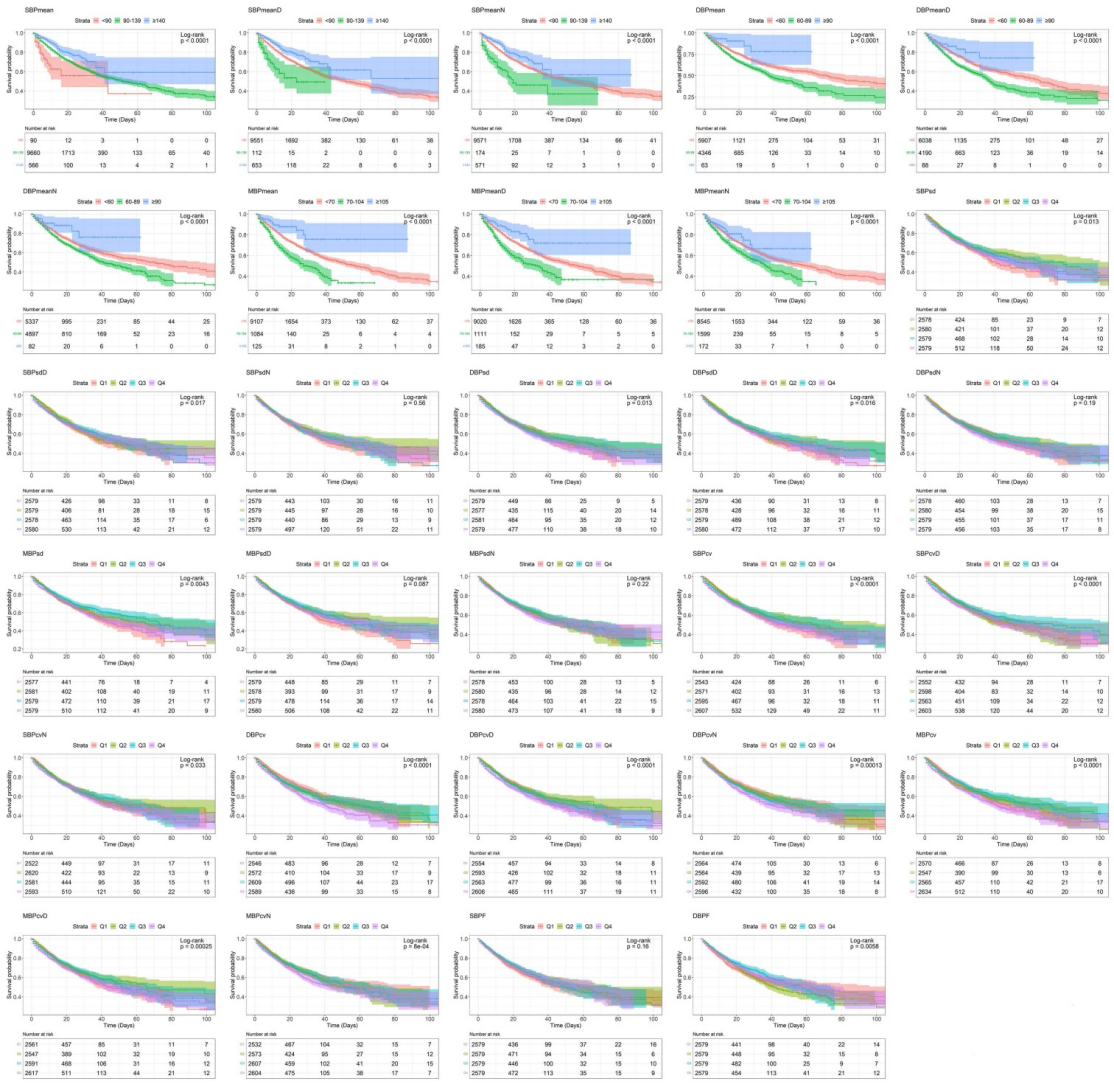
Survivorship curve by Kaplan-Meier method. Abbreviations: SBP, systolic blood pressure; DBP, diastolic blood pressure; MBP, mean blood pressure; SBPF, fluctuations in SBP; DBPF, fluctuations in DBP.

The Kaplan-Meier curves of all examined categorical variables are shown in Figure 1. The log-rank test revealed that all of the examined parameters except SBPsdN, DBPsdN, MBPsdD, MBPsdN, and SBPF were associated with significant survival differences among groups.

The Cox models revealed that before adjusting for covariates, SBPmean, SBPmeanD, and SBPmeanN indicated that the hypotensive group had a higher risk of in-hospital death than the normal blood pressure group, while the hypertensive groups had a lower risk. However, after adjusting all covariates, only the hypotensive group had a significantly higher risk. DBPmean, DBPmeanD, MBPmean, MBPmeanD, and MBPmeanN showed similar results, but DBPmeanN showed a significant increase in risk of in-hospital death only in the hypotensive group before adjustment, while the difference was not statistically significant after adjustment.

SBPsd, SBPsdD, and SBPsdN values in quartile 2 (Q2), quartile 3 (Q3), and quartile (Q4) did not differ from the values in quartile 1 (Q1) before adjustment, and all showed a significantly lower risk after adjustment. SBPcv, SBPcvD, and SBPcvN showed a significantly higher risk only in Q4 before adjustment. However, after adjustment, SBPcv showed a significantly lower risk in Q2 and Q3, SBPcvD showed a significantly lower risk in Q3, and SBPcvN showed a significantly lower risk in Q3 and Q4.

SBPF showed a significantly lower risk in Q3 than in Q1 before adjustment, with no significant difference observed after adjustment. DBPF showed a significantly lower risk in Q3 and Q4 than in Q1 before adjustment, but only in Q3 after adjustment.

### Subgroup analysis of Cox proportional-hazards models

The subgroup analysis revealed that MBPmeanD, MBPmeanN, DBPcvD, and MBPcvD interacted with age, and that MBPmean, MBPmeanN, SBPcv, and SBPcvN interacted with gender. Further stratified analysis showed that MBPmeanD and MBPmeanN had an effect on in-hospital death only among those aged ≥65 years, while DBPcvN and MBPcvD only had an effect among those aged <65 years. Moreover, the effects of MBPmeanN, SBPcv, and SBPcvN were only seen in males, while the effect of MBPmean was more pronounced in males (Table 3).

### RCS analysis

**Figure 2:**
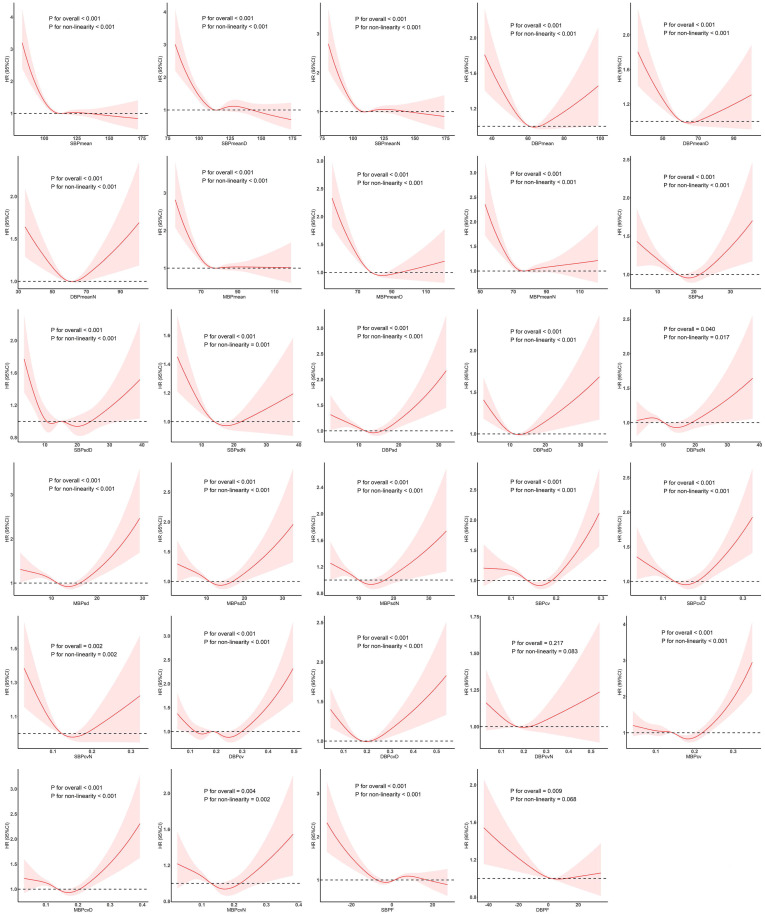
Restricted cubic spline model showing association between examined parameters and the occurrence of in-hospital death. Abbreviations: SBP, systolic blood pressure; DBP, diastolic blood pressure; MBP, mean blood pressure; SBPF, fluctuations in SBP; DBPF, fluctuations in DBP.

The results of the RCS analysis in Figure 2 show that only DBPcvN was not associated with in-hospital death, and all of the examined parameters except DBPF had a nonlinear relationship with the outcome. There were approximate L-shaped dose-response relationships of SBPmean, SBPmeanD, and SBPmeanD with in-hospital death, with a change point at about 110 mmHg. Below that blood pressure, the risk increased as the mean value decreased, and thereafter the effect almost disappeared. DBPmean, DBPmeanD, and DBPmeanN exhibited U-shaped dose-response relationships with the outcome, with a change point at approximately 60 mmHg. The relationships of MBPmean, MBPmeanD, and MBPmeanN with the outcome risk were also roughly L-shaped, with a change point at around 70 mmHg.

SBPsd and SBPsdN showed U-shaped relationships with in-hospital death, with a change point at around 20 mmHg. SBPsdD showed a W-shaped relationship, with change points at around 10, 15, and 20 mmHg, and significant negative and positive correlations were found when the value was lower than 10 mmHg and higher than 20 mmHg, respectively. DBPsd and DBPsdD showed roughly U-shaped relationships, with a change point at around 15 mmHg. DBPsdN showed an inverse N-shaped relationship, with change points at 8 and 15 mmHg. As DBPsdN increased, the risk of in-hospital death first increased and then decreased. MBPsd, MBPsdD, and MBPsdN showed U-shaped dose-response relationships with the outcome, and the change point was at about 15 mmHg.

SBPcv, SBPcvD, and SBPcvN showed U-shaped relationships, with a change point at about 0.15. DBPcv showed a W-shaped relationship, with change points at about 0.15, 0.2, and 0.25, while DBPcvD and DBPcvN showed U-shaped relationships, with a change point at about 0.2. The dose-response relationships of MBPcv, MBPcvD, and MBPcvN with the risk of in-hospital death were roughly U-shaped, with a change point at about 0.2.

SBPF showed an inverse N-shaped relationship with the outcome, with change points at around -5% and 5%, and a significant negative correlation below -5%. DBPF was associated with the risk of in-hospital death, but no significant nonlinear relationship was observed. The negative correlation was more obvious at values below 0%.

### Subgroup analysis of RCS

**Figure 3:**
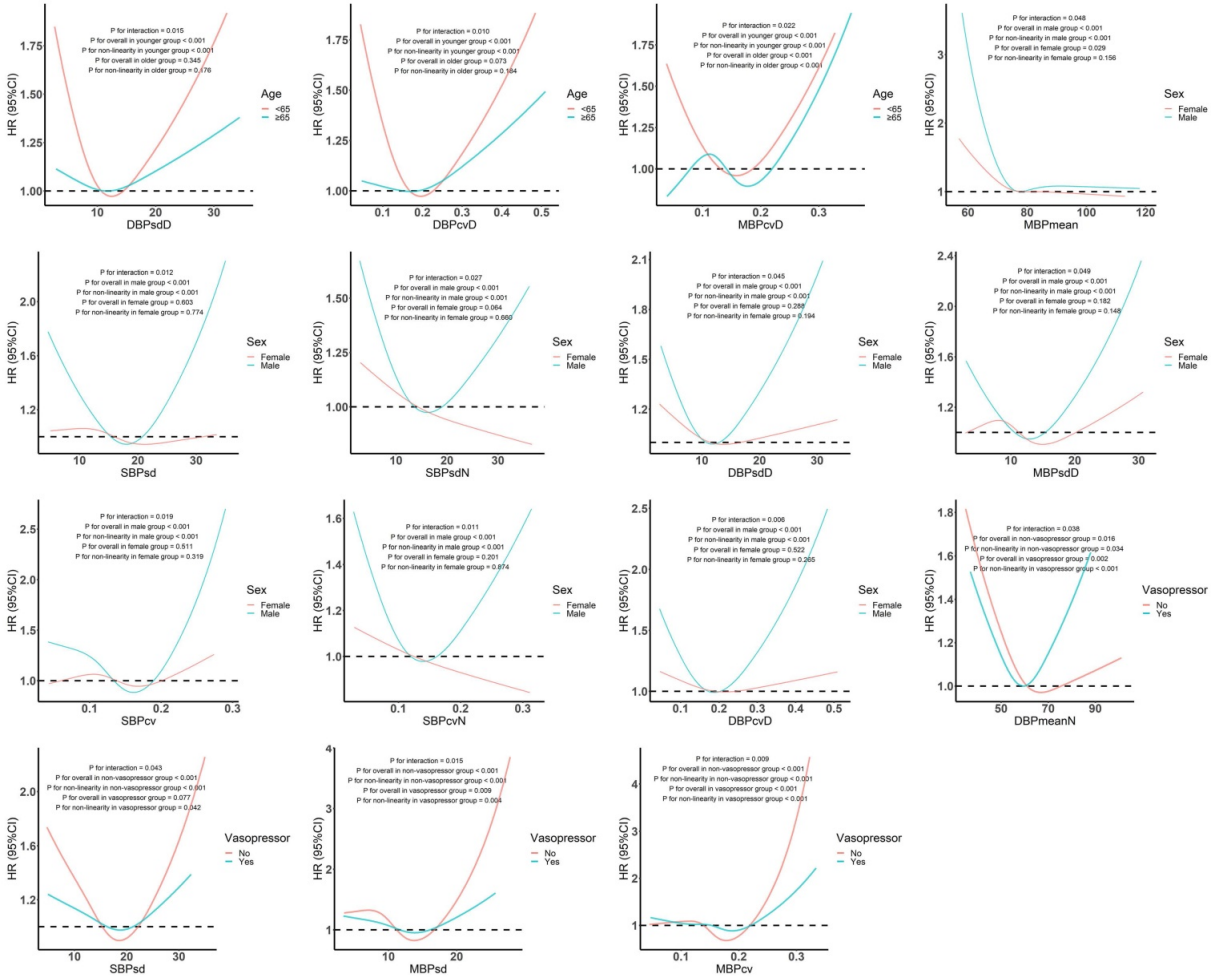
Subgroup analysis of restricted cubic spline model. Abbreviations: SBP, systolic blood pressure; DBP, diastolic blood pressure; MBP, mean blood pressure.

Further interaction analyses (Figure 3) showed that DBPsdD, DBPcvD, and MBPcvD interacted with age. The nonlinear effects of DBPsdD and DBPcvD on in-hospital death were found only among those aged <65 years. The relationship between MBPcvD and outcome was U-shaped among those aged <65 years, and N-shaped among those aged ≥65 years. MBPmean, SBPsd, SBPsdN, DBPsdD, MBPsdD, SBPcv, SBPcvN, and DBPcvD interacted with sex, and the nonlinear correlations between them and in-hospital death only appeared in males. In addition, DBPmeanN, SBPsd, MBPsd, and MBPcv showed different dose-response relationships with the outcome in patients with sepsis and septic shock.

## Discussion

BPV is a very complicated life phenomenon that is affected by numerous factors 3. Although the regulatory mechanisms of BPV remain unclear, its main influencing factors are the nervous system 18, endocrine system, renin-angiotensin-aldosterone system, antidiuretic hormone and other neuroendocrine mechanisms, reflection mechanism (cardiopulmonary reflection), blood viscosity, arterial elasticity, mental state, emotional factors, behavioral factors (e.g., activity, sleep, posture), environmental factors (e.g., long-term exposure to environments with high industrial pollution), lifestyle (exercise, eating habits), the presence of disease, smoking, and drugs 19.

Our analyses of Cox proportional-hazards models showed that SBPmean, SBPmeanD, and SBPmean values <90 mmHg; DBPmean, DBPmeanD, and DBPmeanN values <60 mmHg; and MBPmean, MBPmeanD, and MBPmeanN values <70 mmHg within 24 h are related to a poor prognosis in patients with sepsis. Further RCS analysis showed that SBP, DBP, and MBP had nonlinear relationships with in-hospital death, with change points at 110, 60, and 70 mmHg, respectively. Below the change points, the mortality rate decreased as the blood pressure increased. The underlying mechanism is that blood pressure is lowered due to cytokines and inflammatory substances in patients in the early stage of sepsis 20, and the loss of a large amount of body fluids leads to a decrease in blood pressure. The increase in blood pressure indicates that the patient has received active treatment measures, such as fluid resuscitation, vasopressors, or control of the source of infection 21. The mortality rate of patients showed a downward trend as the blood pressure increased over a certain range.

Our results indicate that variations in SBP, DBP and MBP are associated with mortality regardless of whether they occur over the entire day, during daytime, or during nighttime. In the RCS analysis, SBPsd, DBPsd, and MBPsd showed U-shaped relationships with in-hospital death, with change points at 20, 15, and 15 mmHg, respectively. The coefficients of variation of SBP and MBP showed U-shaped relationships with in-hospital death, and the change points were at around 0.15 and 0.2, respectively. A W-shaped relationship was observed between DBPcv and in-hospital death, and DBPcvD and DBPcvN showed U-shaped relationships with in-hospital death. SBPF had an inverse N-shaped relationship with the risk of in-hospital death, and a negative correlation with the risk of in-hospital death was obvious below -5%. DBPF was related to the risk of in-hospital death, and a negative correlation was obvious below 0%. Healthy people exhibit obvious circadian rhythms in the blood pressure 22, with the blood pressure being higher during the daytime and lower at night. This is related to circadian rhythm changes of the sympathetic nerve and vagus nerve, as well as regulation of the body fluid hormone secretion rhythm in the human body. These changes play an important role in adapting to body activities and protecting cardiovascular structure and function 23. Patients with sepsis will experience abnormal circadian rhythms due to severe damage to the body, neurological and endocrine regulation disorders, and external factors such as drugs, which induce pathological blood pressure variations 24. Sepsis interferes with blood pressure rhythms, and pathological variations occur due to various factors. If the blood pressure exceeds a certain range, it will have an adverse effect on the patient's prognosis. During treatment that leads to low organ perfusion, or when the patient's blood pressure is gradually returning to its own baseline level, the excessive use of vasoactive drugs and other treatments can cause vasoconstriction, which can lead to the kidney and other organs to becoming dysfunctional due to ischemia, resulting in a poor prognosis 25,26. Our results support these mechanisms. Therefore, during the clinical treatment of patients with sepsis, it is necessary to ensure that the blood pressure is maintained, and also that the BPV and circadian rhythm are considered comprehensively in order to improve the prognosis and the survival rate.

The subgroup analysis revealed interactions of DBPsdD, DBPcvD, and MBPcvD with age. Blood pressure is greatly affected by age, with elderly patients having reduced blood vessel elasticity due to problems such as atherosclerosis and vascular sclerosis, allowing the above interactions to occur 27. Moreover, MBPmean, SBPsd, SBPsdN, DBPsdD, MBPsdD, SBPcv, SBPcvN, and DBPcvD interacted with sex, and the nonlinear correlations between these parameters and in-hospital death only appeared in males. Previous studies found that female sex hormones influence vascular function during the menstrual cycle 28,29, and differences in sex-related sympathetic nerve activity may also be an underlying mechanism 30. In addition, DBPmeanN, SBPsd, MBPsd, and MBPcv showed different dose-response relationships in patients with sepsis and septic shock. The most-fundamental difference between sepsis and septic shock is that in septic shock, vasopressors are used to maintain the mean arterial pressure. Therefore, treatment is the main reason for the differences in the effects of DBPmeanN, SBPsd, MBPsd and MBPcv on these two subgroups 21.

## Strengths and limitations

Most previous studies have focused on the effects of blood pressure and its variability in patients with hypertension or in healthy people on the heart, brain, and other organs, with few studies investigating the effects of blood pressure and its variability on prognosis in sepsis patients 31,32. To our knowledge, the present study is the first to analyze a large critical-illness database in order to identify the predictive value of indicators of the ambulatory blood pressure on in-hospital death in sepsis patients. Moreover, a detailed analysis was performed of the average blood pressure, BPV, and circadian rhythm indicators to provide a basis for the clinical treatment of patients with sepsis.

Of course, this study was also subject to certain limitations. First, it had a retrospective design, and the MIMIC-IV data coming from a single center may have resulted in selection bias. These two aspects restrict the ability to extrapolate our results, and so they need to be validated in a prospective cohort from multiple centers. Second, imputing missing values will lead to a certain degree of information bias. Third, some potential confounding factors such as the specific dosage of vasopressor were not taken into account due to the limitations of the database. Fourth, the present research would have benefited from a more regular measurement of blood pressure.

## Conclusion

Indicators of the ambulatory blood pressure measured within 24 h are related to the prognosis of sepsis patients. When treating patients with sepsis, in addition to measuring blood pressure, attention should be paid to their BPV and circadian rhythm indicators in order to improve the prognosis and the survival rate.

## Figures and Tables

**Table 1:** Baseline characteristics

Variable	Overall	Alive	Dead	P-value
N	10316	8199	2117	
Age, years	67.00 [56.00, 78.00]	66.00 [55.00, 78.00]	70.00 [59.00, 80.00]	<0.001
**Sex (%)**				
Male	5312 (51.5)	4202 (51.3)	1110 (52.4)	0.344
Female	5004 (48.5)	3997 (48.7)	1007 (47.6)	
**Ethnicity (%)**				
White	6985 (67.7)	5630 (68.7)	1355 (64.0)	<0.001
Black	1205 (11.7)	997 (12.2)	208 (9.8)	
Other	2126 (20.6)	1572 (19.2)	554 (26.2)	
Weight, kg	77.05 [64.60, 93.60]	77.30 [64.70, 94.00]	76.10 [64.20, 92.50]	0.023
Charlson Comorbidity Index	3.00 [1.00, 5.00]	3.00 [1.00, 5.00]	4.00 [2.00, 6.00]	<0.001
**Myocardial infarct (%)**				
No	8709 (84.4)	6997 (85.3)	1712 (80.9)	<0.001
Yes	1607 (15.6)	1202 (14.7)	405 (19.1)	
**Congestive heart failure (%)**				
No	7062 (68.5)	5685 (69.3)	1377 (65.0)	<0.001
Yes	3254 (31.5)	2514 (30.7)	740 (35.0)	
**Cerebrovascular disease (%)**				
No	8858 (85.9)	7099 (86.6)	1759 (83.1)	<0.001
Yes	1458 (14.1)	1100 (13.4)	358 (16.9)	
**Renal disease (%)**				
No	7570 (73.4)	6089 (74.3)	1481 (70.0)	<0.001
Yes	2746 (26.6)	2110 (25.7)	636 (30.0)	
**Mild liver disease (%)**				
No	8665 (84.0)	7078 (86.3)	1587 (75.0)	<0.001
Yes	1651 (16.0)	1121 (13.7)	530 (25.0)	
**Severe liver disease (%)**				
No	9442 (91.5)	7657 (93.4)	1785 (84.3)	<0.001
Yes	874 (8.5)	542 (6.6)	332 (15.7)	
**Uncomplicated diabetes (%)**				
No	7770 (75.3)	6138 (74.9)	1632 (77.1)	0.037
Yes	2546 (24.7)	2061 (25.1)	485 (22.9)	
**Complicated diabetes (%)**				
No	9124 (88.4)	7234 (88.2)	1890 (89.3)	0.192
Yes	1192 (11.6)	965 (11.8)	227 (10.7)	
**Malignant cancer (%)**				
No	8927 (86.5)	7223 (88.1)	1704 (80.5)	<0.001
Yes	1389 (13.5)	976 (11.9)	413 (19.5)	
SOFA	3.00 [2.00, 4.00]	3.00 [2.00, 4.00]	3.00 [3.00, 5.00]	<0.001
APSIII	55.00 [42.00, 73.00]	51.00 [39.00, 66.00]	78.00 [61.00, 98.00]	<0.001
Lactate (mmol/L)	1.80 [1.20, 2.70]	1.70 [1.20, 2.50]	2.20 [1.40, 3.70]	<0.001
Creatinine (mg/dL)	1.20 [0.80, 2.00]	1.10 [0.80, 1.90]	1.40 [0.90, 2.40]	<0.001
WBC (k/uL)	11.60 [7.80, 16.60]	11.30 [7.70, 16.10]	12.40 [8.00, 18.40]	<0.001
**Ventilation (%)**				
No	3659 (35.5)	3325 (40.6)	334 (15.8)	<0.001
Yes	6657 (64.5)	4874 (59.4)	1783 (84.2)	
**Vasopressor (%)**				
No	5885 (57.0)	5197 (63.4)	688 (32.5)	<0.001
Yes	4431 (43.0)	3002 (36.6)	1429 (67.5)	
**CRRT (%)**				
No	9662 (93.7)	7890 (96.2)	1772 (83.7)	<0.001
Yes	654 (6.3)	309 (3.8)	345 (16.3)	
SBPmean, mmHg	113.00 [106.00, 120.00]	113.00 [106.00, 121.00]	111.00 [104.00, 118.00]	<0.001
SBPmeanD, mmHg	113.00 [106.00, 122.00]	114.00 [106.00, 123.00]	111.00 [104.00, 119.00]	<0.001
SBPmeanN, mmHg	112.00 [104.00, 121.00]	112.00 [104.50, 121.00]	110.00 [102.00, 118.00]	<0.001
DBPmean, mmHg	61.00 [56.00, 65.00]	61.00 [57.00, 66.00]	60.00 [55.00, 64.00]	<0.001
DBPmeanD, mmHg	61.00 [56.00, 67.00]	62.00 [57.00, 67.00]	60.00 [55.00, 65.00]	<0.001
DBPmeanN, mmHg	60.00 [55.00, 65.00]	60.00 [55.00, 66.00]	59.00 [54.00, 65.00]	<0.001
MBPmean, mmHg	78.00 [74.00, 83.00]	78.00 [74.00, 83.00]	77.00 [72.00, 81.00]	<0.001
MBPmeanD, mmHg	78.00 [74.00, 84.00]	79.00 [74.00, 85.00]	77.00 [72.00, 82.00]	<0.001
MBPmeanN, mmHg	77.00 [72.00, 83.00]	77.00 [72.00, 83.00]	76.00 [71.00, 82.00]	<0.001
SBPsd, mmHg	15.66 [11.65, 19.78]	15.55 [11.64, 19.62]	16.08 [11.68, 20.53]	0.001
SBPsdD, mmHg	14.77 [10.77, 19.46]	14.61 [10.75, 19.29]	15.45 [10.80, 20.10]	<0.001
SBPsdN, mmHg	13.98 [9.58, 19.31]	13.93 [9.61, 19.18]	14.25 [9.37, 19.64]	0.279
DBPsd, mmHg	11.59 [8.65, 14.48]	11.52 [8.61, 14.39]	11.89 [8.76, 14.92]	0.004
DBPsdD, mmHg	11.12 [7.93, 14.38]	11.05 [7.93, 14.24]	11.51 [7.93, 14.84]	0.011
DBPsdN, mmHg	10.28 [6.83, 14.20]	10.20 [6.79, 14.13]	10.60 [6.93, 14.39]	0.027
MBPsd, mmHg	11.37 [8.63, 14.20]	11.34 [8.62, 14.08]	11.51 [8.67, 14.71]	0.003
MBPsdD, mmHg	10.88 [7.90, 13.99]	10.81 [7.90, 13.89]	11.17 [7.89, 14.48]	0.003
MBPsdN, mmHg	10.14 [6.98, 13.88]	10.09 [6.96, 13.78]	10.38 [7.07, 14.16]	0.080
SBPcv	0.14 [0.10, 0.17]	0.13 [0.10, 0.17]	0.14 [0.11, 0.18]	<0.001
SBPcvD	0.13 [0.10, 0.17]	0.13 [0.10, 0.16]	0.14 [0.10, 0.18]	<0.001
SBPcvN	0.12 [0.09, 0.17]	0.12 [0.09, 0.17]	0.13 [0.09, 0.18]	0.002
DBPcv	0.19 [0.14, 0.24]	0.19 [0.14, 0.24]	0.20 [0.15, 0.25]	<0.001
DBPcvD	0.18 [0.13, 0.23]	0.18 [0.13, 0.23]	0.19 [0.13, 0.25]	<0.001
DBPcvN	0.17 [0.12, 0.23]	0.17 [0.11, 0.23]	0.18 [0.12, 0.24]	<0.001
MBPcv	0.14 [0.11, 0.18]	0.14 [0.11, 0.18]	0.15 [0.12, 0.19]	<0.001
MBPcvD	0.14 [0.10, 0.18]	0.14 [0.10, 0.17]	0.14 [0.10, 0.18]	<0.001
MBPcvN	0.13 [0.09, 0.18]	0.13 [0.09, 0.17]	0.13 [0.09, 0.18]	<0.001
SBPF (%)	1.31 [-4.25, 6.62]	1.37 [-4.10, 6.61]	1.12 [-4.78, 6.70]	0.293
DBPF (%)	2.13 [-4.81, 8.58]	2.32 [-4.58, 8.65]	1.26 [-5.73, 8.30]	0.004
Length of stay in hospital,day	10.00 [6.00, 20.00]	10.00 [6.00, 20.00]	11.00 [5.00, 21.00]	0.003

Abbreviations: SOFA, Sequential Organ Failure Assessment; APSIII, Acute Physiological Scores III; WBC, white blood cell; CRRT, continuous renal replacement therapy; SBP, systolic blood pressure; DBP, diastolic blood pressure; MBP, mean blood pressure; SBPF, fluctuations in SBP; DBPF, fluctuations in DBP.

**Table 2:** Results of Cox proportional-hazards models

Variable	Group	No. of death/No. of patients (Incidence rate)	Unadjusted model		Adjusted model	
			HR (95%CI)	P-value	HR (95%CI)	P-value
SBPmean (mmHg)	Continuous			<0.001		<0.001
	<90	31/90(34.4%)	1.956(1.372-2.789)	<0.001	2.346(1.594-3.451)	<0.001
	90-139	2010/9660(20.8%)	Reference		Reference	
	≥140	76/566(13.4%)	0.637(0.507-0.802)	<0.001	0.880(0.674-1.148)	0.345
SBPmeanD (mmHg)	Continuous			<0.001		<0.001
	<90	41/112(36.6%)	2.122(1.558-2.892)	<0.001	2.050(1.445-2.908)	<0.001
	90-139	1986/9551(20.8%)	Reference		Reference	
	≥140	90/653(13.8%)	0.636(0.515-0.785)	<0.001	0.809(0.634-1.033)	0.089
SBPmeanN(mmHg)	Continuous			<0.001		<0.001
	<90	67/174(38.5%)	2.153(1.688-2.747)	<0.001	2.159(1.661-2.804)	<0.001
	90-139	1969/9571(20.6%)	Reference		Reference	
	≥140	81/571(14.2%)	0.694(0.555-0.866)	0.001	0.881(0.681-1.140)	0.334
DBPmean (mmHg)	Continuous			<0.001		0.026
	<60	1019/4346(23.4%)	1.398(1.283-1.523)	<0.001	1.106(1.009-1.213)	0.031
	60-89	1091/5907(18.5%)	Reference		Reference	
	≥90	7/63(11.1%)	0.476(0.226-1.001)	0.050	0.859(0.407-1.813)	0.690
DBPmeanD (mmHg)	Continuous			<0.001		0.005
	<60	1003/4190(23.9%)	1.436(1.318-1.564)	<0.001	1.154(1.053-1.263)	0.002
	60-89	1101/6038(18.2%)	Reference		Reference	
	≥90	13/88(14.8%)	0.660(0.382-1.140)	0.136	1.191(0.655-2.167)	0.566
DBPmeanN (mmHg)	Continuous			<0.001		0.376
	<60	1107/4897(22.6%)	1.267(1.163-1.380)	<0.001	1.039(0.948-1.138)	0.413
	60-89	1000/5337(18.7%)	Reference		Reference	
	≥90	10/82(12.2%)	0.555(0.297-1.034)	0.064	0.966(0.499-1.869)	0.918
MBPmean (mmHg)	Continuous			<0.001		<0.001
	<70	307/1084(28.3%)	1.672(1.481-1.888)	<0.001	1.472(1.293-1.676)	<0.001
	70-104	1795/9107(19.7%)	Reference		Reference	
	≥105	15/125(12.0%)	0.507(0.305-0.843)	0.009	0.935(0.550-1.589)	0.804
MBPmeanD (mmHg)	Continuous			<0.001		<0.001
	<70	313/1111(28.2%)	1.617(1.433-1.823)	<0.001	1.385(1.218-1.575)	<0.001
	70-104	1779/9020(19.9%)	Reference		Reference	
	≥105	25/185(13.5%)	0.586(0.395-0.870)	0.008	1.021(0.668-1.561)	0.923
MBPmeanN (mmHg)	Continuous			<0.001		0.010
	<70	413/1599(25.8%)	1.440(1.293-1.603)	<0.001	1.247(1.112-1.398)	<0.001
	70-104	1676/8545(19.6%)	Reference		Reference	
	≥105	28/172(16.3%)	0.780(0.537-1.133)	0.192	1.282(0.860-1.911)	0.223
SBPsd (mmHg)	Continuous			0.016		0.056
	Q1(<11.653)	523/2578(20.3%)	Reference		Reference	
	Q2(11.653-15.660)	474/2580(18.4%)	0.913(0.806-1.034)	0.153	0.824(0.723-0.939)	0.004
	Q3(15.660-19.777)	507/2579(19.7%)	0.958(0.848-1.082)	0.488	0.779(0.684-0.887)	<0.001
	Q4(>19.777)	613/2579(23.8%)	1.104(0.982-1.241)	0.097	0.848(0.748-0.960)	0.009
SBPsdD (mmHg)	Continuous			0.020		0.072
	Q1(<10.768)	524/2579(20.3%)	Reference		Reference	
	Q2(10.768-14.775)	451/2579(17.5%)	0.885(0.780-1.004)	0.058	0.789(0.691-0.901)	<0.001
	Q3(14.775-19.456)	543/2578(21.1%)	1.038(0.920-1.170)	0.546	0.840(0.739-0.954)	0.007
	Q4(>19.456)	599/2580(23.2%)	1.072(0.953-1.205)	0.247	0.810(0.715-0.918)	<0.001
SBPsdN (mmHg)	Continuous			0.251		0.012
	Q1(<9.576)	524/2579(20.3%)	Reference		Reference	
	Q2(9.576-13.982)	451/2579(17.5%)	0.936(0.828-1.057)	0.287	0.843(0.742-0.957)	0.009
	Q3(13.982-19.309)	543/2578(21.1%)	0.985(0.874-1.111)	0.808	0.853(0.752-0.968)	0.014
	Q4(>19.309)	599/2580(23.2%)	1.020(0.906-1.147)	0.747	0.789(0.696-0.894)	<0.001
DBPsd (mmHg)	Continuous			0.002		0.662
	Q1(<8.648)	506/2579(19.6%)	Reference		Reference	
	Q2(8.648-11.586)	495/2577(19.2%)	0.988(0.872-1.118)	0.845	0.953(0.837-1.085)	0.469
	Q3(11.586-14.485)	516/2581(20.0%)	1.034(0.915-1.169)	0.590	0.859(0.754-0.978)	0.021
	Q4(>14.485)	600/2579(23.3%)	1.176(1.045-1.324)	0.007	0.945(0.833-1.072)	0.383
DBPsdD (mmHg)	Continuous			0.024		0.479
	Q1(<7.929)	529/2579(20.5%)	Reference		Reference	
	Q2(7.929-11.124)	474/2578(18.4%)	0.893(0.789-1.011)	0.075	0.870(0.765-0.991)	0.035
	Q3(11.124-14.376)	523/2579(20.3%)	0.964(0.855-1.088)	0.556	0.809(0.712-0.918)	0.001
	Q4(>14.376)	591/2580(22.9%)	1.085(0.965-1.220)	0.174	0.911(0.804-1.031)	0.138
DBPsdN (mmHg)	Continuous			0.004		0.588
	Q1(<6.831)	511/2578(19.8%)	Reference		Reference	
	Q2(6.831-10.278)	509/2580(19.7%)	1.009(0.892-1.140)	0.890	1.013(0.892-1.151)	0.843
	Q3(10.278-14.196)	542/2579(21.0%)	1.081(0.957-1.220)	0.209	0.964(0.849-1.095)	0.575
	Q4(>14.196)	555/2579(21.5%)	1.121(0.994-1.264)	0.063	0.886(0.781-1.005)	0.061
MBPsd (mmHg)	Continuous			0.005		0.241
	Q1(<8.628)	524/2579(20.3%)	Reference		Reference	
	Q2(8.628-11.372)	505/2579(19.6%)	0.982(0.869-1.110)	0.773	0.880(0.773-1.001)	0.051
	Q3(11.372-14.198)	476/2579(18.5%)	0.893(0.789-1.011)	0.074	0.748(0.656-0.852)	<0.001
	Q4(>14.198)	612/2579(23.7%)	1.112(0.990-1.250)	0.075	0.857(0.757-0.971)	0.015
MBPsdD (mmHg)	Continuous			0.019		0.214
	Q1(<7.898)	530/2579(20.6%)	Reference		Reference	
	Q2(7.898-10.876)	481/2579(18.7%)	0.941(0.831-1.064)	0.332	0.912(0.801-1.037)	0.161
	Q3(10.876-13.995)	506/2579(19.6%)	0.949(0.840-1.072)	0.401	0.791(0.696-0.899)	<0.001
	Q4(>13.995)	600/2579(23.3%)	1.079(0.960-1.213)	0.204	0.855(0.755-0.968)	0.013
MBPsdN (mmHg)	Continuous			0.031		0.114
	Q1(<6.976)	519/2579(20.1%)	Reference		Reference	
	Q2(6.976-10.136)	506/2579(19.6%)	0.999(0.884-1.129)	0.987	0.931(0.820-1.058)	0.274
	Q3(10.136-13.884)	522/2579(20.2%)	1.008(0.892-1.138)	0.899	0.824(0.725-0.936)	0.003
	Q4(>13.884)	570/2579(22.1%)	1.111(0.986-1.251)	0.084	0.890(0.786-1.008)	0.067
SBPcv	Continuous			<0.001		0.759
	Q1(<0.104)	487/2543(19.2%)	Reference		Reference	
	Q2(0.104-0.136)	454/2571(17.7%)	0.949(0.835-1.078)	0.421	0.866(0.757-0.991)	0.036
	Q3(0.136-0.172)	523/2595(20.2%)	1.069(0.945-1.209)	0.292	0.833(0.731-0.950)	0.006
	Q4(>0.172)	653/2607(25.0%)	1.238(1.101-1.393)	<0.001	0.891(0.786-1.011)	0.073
SBPcvD	Continuous			<0.001		0.852
	Q1(<0.096)	475/2552(18.6%)	Reference		Reference	
	Q2(0.096-0.129)	481/2598(18.5%)	1.043(0.919-1.184)	0.516	0.910(0.796-1.040)	0.168
	Q3(0.129-0.168)	503/2563(19.6%)	1.052(0.928-1.193)	0.428	0.842(0.737-0.962)	0.011
	Q4(>0.168)	658/2603(25.3%)	1.292(1.148-1.454)	<0.001	0.928(0.818-1.053)	0.245
SBPcvN	Continuous			<0.001		0.107
	Q1(<0.086)	505/2522(20.0%)	Reference		Reference	
	Q2(0.086-0.123)	491/2620(18.7%)	0.994(0.878-1.126)	0.925	0.895(0.786-1.020)	0.096
	Q3(0.123-0.169)	517/2581(20.0%)	1.042(0.922-1.178)	0.509	0.868(0.763-0.987)	0.031
	Q4(>0.169)	604/2593(23.3%)	1.161(1.031-1.306)	0.014	0.852(0.751-0.966)	0.012
DBPcv	Continuous			<0.001		0.619
	Q1(<0.142)	456/2546(17.9%)	Reference		Reference	
	Q2(0.142-0.190)	499/2572(19.4%)	1.165(1.026-1.323)	0.019	0.996(0.872-1.138)	0.952
	Q3(0.190-0.239)	533/2609(20.4%)	1.163(1.026-1.318)	0.018	0.890(0.780-1.017)	0.086
	Q4(>0.239)	629/2589(24.3%)	1.448(1.284-1.634)	<0.001	0.981(0.862-1.116)	0.770
DBPcvD	Continuous			<0.001		0.661
	Q1(<0.130)	497/2554(19.5%)	Reference		Reference	
	Q2(0.130-0.181)	463/2593(17.9%)	0.945(0.832-1.072)	0.380	0.860(0.753-0.983)	0.026
	Q3(0.181-0.235)	523/2563(20.4%)	1.052(0.930-1.189)	0.422	0.851(0.748-0.968)	0.014
	Q4(>0.235)	634/2606(24.3%)	1.267(1.127-1.425)	<0.001	0.920(0.811-1.042)	0.190
DBPcvN	Continuous			<0.001		0.820
	Q1(<0.115)	481/2564(18.8%)	Reference		Reference	
	Q2(0.115-0.171)	493/2564(19.2%)	1.074(0.947-1.218)	0.265	0.977(0.857-1.113)	0.724
	Q3(0.171-0.235)	549/2592(21.2%)	1.152(1.019-1.303)	0.023	0.944(0.829-1.074)	0.382
	Q4(>0.235)	594/2596(22.9%)	1.302(1.155-1.469)	<0.001	0.940(0.827-1.068)	0.340
MBPcv	Continuous			<0.001		0.963
	Q1(<0.111)	484/2579(18.8%)	Reference		Reference	
	Q2(0.111-0.144)	495/2579(19.2%)	1.110(0.979-1.259)	0.105	0.951(0.833-1.086)	0.461
	Q3(0.144-0.179)	485/2579(18.8%)	1.034(0.911-1.173)	0.607	0.822(0.719-0.939)	0.004
	Q4(>0.179)	653/2579(25.3%)	1.344(1.195-1.511)	<0.001	0.910(0.801-1.033)	0.145
MBPcvD	Continuous			<0.001		0.919
	Q1(<0.101)	503/2579(19.5%)	Reference		Reference	
	Q2(0.101-0.137)	471/2579(18.3%)	0.976(0.860-1.108)	0.711	0.929(0.814-1.060)	0.276
	Q3(0.137-0.176)	508/2579(19.7%)	1.014(0.896-1.147)	0.827	0.806(0.707-0.919)	0.001
	Q4(>0.176)	635/2579(24.6%)	1.224(1.089-1.376)	<0.001	0.908(0.802-1.028)	0.126
MBPcvN	Continuous			<0.001		0.253
	Q1(<0.091)	510/2579(19.8%)	Reference		Reference	
	Q2(0.091-0.129)	482/2579(18.7%)	1.006(0.888-1.140)	0.925	0.926(0.813-1.055)	0.248
	Q3(0.129-0.177)	522/2579(20.2%)	1.049(0.928-1.186)	0.443	0.824(0.725-0.937)	0.003
	Q4(>0.177)	603/2579(23.4%)	1.235(1.097-1.390)	<0.001	0.908(0.801-1.029)	0.130
SBPF (%)	Continuous			0.022		0.084
	Q1(<-4.250)	572/2579(22.2%)	Reference		Reference	
	Q2(-4.250-1.306)	507/2579(19.7%)	0.890(0.790-1.004)	0.057	0.924(0.816-1.047)	0.214
	Q3(1.306-6.625)	501/2579(19.4%)	0.885(0.785-0.998)	0.047	0.959(0.846-1.087)	0.510
	Q4(>6.625)	537/2579(20.8%)	0.925(0.822-1.041)	0.197	0.950(0.840-1.074)	0.410
DBPF (%)	Continuous			<0.001		0.017
	Q1(<-4.806)	585/2579(22.7%)	Reference		Reference	
	Q2(-4.806-2.135)	536/2579(20.8%)	0.902(0.802-1.014)	0.085	0.963(0.852-1.088)	0.546
	Q3(2.135-8.583)	483/2579(18.7%)	0.811(0.719-0.915)	<0.001	0.879(0.775-0.996)	0.043
	Q4(>8.583)	513/2579(19.9%)	0.866(0.769-0.974)	0.017	0.915(0.809-1.034)	0.155

Abbreviations: SBP, systolic blood pressure; DBP, diastolic blood pressure; MBP, mean blood pressure; SBPF, fluctuations in SBP; DBPF, fluctuations in DBP.

**Table 3:** Results of subgroup analysis

Variable	Age		P for interaction	Variable	Sex		P for interaction
	<65	≥65			Male	Female	
**MBPmeanD**			0.042	**MBPmean**			0.041
<70	1.184(0.942-1.489)	1.559(1.333-1.824)		<70	1.765(1.475-2.112)	1.223(1.012-1.479)	
50-104	Reference	Reference		50-104	Reference	Reference	
≥105	0.736(0.391-1.384)	1.182(0.666-2.100)		≥105	0.915(0.454-1.845)	0.935(0.415-2.108)	
**MBPmeanN**			0.016	**MBPmeanN**			0.040
<70	1.139(0.936-1.387)	1.401(1.216-1.614)		<70	1.408(1.198-1.655)	1.097(0.930-1.295)	
50-104	Reference	Reference		50-104	Reference	Reference	
≥105	0.791(0.407-1.540)	1.760(1.067-2.903)		≥105	1.671(1.028-2.716)	0.858(0.424-1.736)	
**DBPcvD**			0.004	**SBPcv**			0.038
Q1	Reference	Reference		Q1	Reference	Reference	
Q2	0.679(0.550-0.838)	1.066(0.894-1.271)		Q2	0.764(0.636-0.918)	1.001(0.818-1.224)	
Q3	0.698(0.567-0.859)	1.014(0.855-1.201)		Q3	0.708(0.590-0.849)	1.001(0.824-1.215)	
Q4	0.819(0.671-0.999)	1.069(0.905-1.262)		Q4	0.842(0.711-0.996)	0.954(0.788-1.156)	
**MBPcvD**			0.012	**SBPcvN**			0.017
Q1	Reference	Reference		Q1	Reference	Reference	
Q2	0.768(0.622-0.948)	1.097(0.923-1.305)		Q2	0.822(0.689-0.982)	1.003(0.826-1.219)	
Q3	0.658(0.530-0.816)	0.953(0.804-1.129)		Q3	0.750(0.626-0.898)	1.015(0.839-1.228)	
Q4	0.855(0.706-1.036)	1.011(0.857-1.193)		Q4	0.868(0.733-1.027)	0.852(0.703-1.031)	

Abbreviations: SBP, systolic blood pressure; DBP, diastolic blood pressure; MBP, mean blood pressure.
